# Relationship between dietary inflammatory index and risk of gestational diabetes *mellitus*: a systematic review

**DOI:** 10.61622/rbgo/2025rbgo34

**Published:** 2025-09-08

**Authors:** Lucas Almeida das Chagas, Rosângela Maria Lopes de Sousa, Ana Vitória Almeida das Chagas da Silva, Luiz Gonzaga Ribeiro Silva-Neto, Bianca de Almeida-Pititto, Larissa Keren de Azevedo Teixeira, Edward Araujo Júnior, Rosiane Mattar

**Affiliations:** 1 Universidade Federal de São Paulo Escola Paulista de Medicina Departamento de Obstetrícia São Paulo SP Brazil Departamento de Obstetrícia, Escola Paulista de Medicina, Universidade Federal de São Paulo, São Paulo, SP, Brazil.; 2 Universidade CEUMA Departamento de Nutrição São Luís MA Brazil Departamento de Nutrição, Universidade CEUMA, São Luís, MA, Brazil.; 3 Faculdade Diferencial Integral Departamento de Farmácia Teresina PI Brazil Departamento de Farmácia, Faculdade Diferencial Integral, Teresina, PI, Brazil.; 4 Universidade Federal de São Paulo Escola Paulista de Medicina Departamento de Nutrição São Paulo SP Brazil Departamento de Nutrição, Escola Paulista de Medicina, Universidade Federal de São Paulo, São Paulo, SP, Brazil.; 5 Universidade Federal de São Paulo Escola Paulista de Medicina Departamento de Medicina Preventiva São Paulo SP Brasil Departamento de Medicina Preventiva, Escola Paulista de Medicina, Universidade Federal de São Paulo, São Paulo, SP, Brasil.; 6 Universidade Municipal de São Caetano do Sul São Caetano do Sul SP Brazil Disciplina de Saúde da Mulher, Universidade Municipal de São Caetano do Sul, São Caetano do Sul, SP, Brazil.

**Keywords:** Inflammation, Diet, Pregnant people, Diabetes, gestational, Diabetes *mellitus*

## Abstract

**Objective:**

To conduct a systematic review of the literature, compiling the available evidence from the last decade to better understand the relationship between the Dietary Inflammatory Index and the risk of Gestational Diabetes Mellitus.

**Methods:**

A comprehensive search was systematically conducted including cohort and case-control studies, researched from the BVS, PubMed, Embase, and Google Scholar platforms with articles published between 2014 and 2024. The risk of bias was assessed using the Quality Assessment Tool.

**Results:**

This review included 10 studies from five countries with participant numbers ranging from 164 to 90,740. In total, four case-control studies found higher DII values in the groups of pregnant women diagnosed with Gestational Diabetes Mellitus compared to those without the diagnosis. When analyzing the remaining cohort studies, one study showed a higher distribution of Gestational Diabetes Mellitus in the highest tertile of Dietary Inflammatory Index, and the other (five studies) showed an association between DII and Gestational Diabetes Mellitus risk when using models adjusted for anthropometry, gestational history, and dietary intake.

**Conclusion:**

Pregnant women diagnosed with Gestational Diabetes Mellitus have a diet with more pro-inflammatory characteristics, which increases the risk of adverse perinatal outcomes.

**Registry of Systematic Reviews (Prospero):**

CRD42024573560

## Introduction

Pregnancy is considered a controlled inflammatory state.^(1)^ However, the presence of overweight and obesity during pregnancy are associated with increased activation of inflammatory cytokines and the emergence of pregnancy-related diseases, among which Gestational Diabetes Mellitus (GDM) is notable.^(2)^

Gestational Diabetes Mellitus is defined as any degree of carbohydrate intolerance of varying severity, diagnosed for the first-time during pregnancy, and it may or may not persist after childbirth.^(3)^ As one of the most common complications in pregnancy, the global prevalence of GDM is estimated to range from 8.4% to 30.9%.^(4,5)^ This variation is the result of differences in the characteristics of the studied population (such as age and obesity) and the diagnostic tests used.^(4,5)^ In Brazil, the prevalence of GDM is 18% according to the criteria of the International Association of Diabetes and Pregnancy Study Groups.^(6,7)^

The increasing incidence of GDM cases worldwide raises concerns for the entire healthcare system.^(8)^ Women diagnosed with GDM have an elevated risk of developing GDM in future pregnancies and an increased long term risk Type 2 Diabetes Mellitus (T2DM). Additionally, the presence of this condition represents higher risks of complications such as stillbirth, preterm birth, macrosomia, hyperinsulinemia, and clinical signs of neonatal hypoglycemia.^(9)^

Although the etiology of GDM is not fully understood, some factors are considered risk predictors for the disease, including maternal age, overweight or obesity, genetic factors, a history of GDM in previous pregnancies, lack of physical activity, and inadequate food consumption.^(10)^

In recent years, the role of diet as a significant component contributing to metabolic disorders has been investigated.^(11,12)^ Diets characterized by the consumption of red meats, processed foods, high-fat dairy products, refined grains, and sweets are related to increased inflammation and oxidative stress in the body, which in turn is associated with a higher risk of GDM.^(13,14)^ This inflammation, resulting from oxidative stress, leads to an increased inflammatory response, implicating the activation of nuclear factor kappa-B (NF-kB) signaling, which is responsible for dysregulating inflammatory pathways and producing elevated levels of pro-inflammatory mediators (such as C-reactive protein (CRP), interleukins, and tumor necrosis factor alpha (TNF-a).^(15)^ On the other hand, diets with anti-inflammatory and antioxidant properties, primarily found in fruits, vegetables, whole grains, nuts, and fish (mediterranean diet), can reduce inflammatory conditions and prevent chronic diseases and GDM.^(16,17)^

In the past decade, there has been growing interest in evaluating the relationship between the general inflammatory effects of a diet through the Dietary Inflammatory Index (DII), which is a validated tool capable of comprehensively assessing the anti-inflammatory and pro-inflammatory effects of an individual's diet based on inflammatory markers such as interleukin-6 (IL-6), TNF-a, and CRP.^(18)^

Although some studies have investigated the relationship between the DII score and the risk of women developing GDM, the results have been scarce and contradictory. Previous investigations found a positive correlation between a higher DII score and an increased risk of women developing GDM during pregnancy.^(16,19)^ These results differ from those found by other researchers who reported no association between the DII and GDM risk.^(20,21)^

Based on these literary findings, it is important to assess more broadly whether an inflammatory diet can be a determining factor in the risk of women developing GDM. Therefore, the objective of this study was to conduct a systematic review of the literature, compiling the available evidence from the past decade to better understand the relationship between the DII and the risk of GDM.

## Methods

This is a systematic review. The protocol for this systematic review was developed following the PRISMA-P method (Preferred Reporting Items for Systematic Reviews and Meta-Analyses – Protocols) and was registered in the Prospective International Registry of Systematic Reviews (Prospero) under the number CRD42024573560.

In this review, cohort and case-control studies published hat evaluated the presence of GDM and the DII of the included pregnant women were included. Studies with the following designs were not included: clinical trials, case reports, reviews, and discussion articles, as well as abstracts, letters to the editor, conference summaries, personal opinions, books, and/or book chapters.

Two examiners were involved in the search and analysis of eligible articles. There was no exchange of information between the two examiners during the article selection process.

The articles were searched on the platforms BVS, PubMed, Embase, and Google Scholar, considering the entire collection, using the descriptors "Gestational Diabetes Mellitus" and "Dietary Inflammatory Index", in combination with keywords combined with Boolean operators AND and OR (Chart 1).

**Chart 1:** Search strategy

Database	Search strategy
PUBMED	Gestational Diabetes Mellitus and Dietary Inflammatory Index,,in the last 10 years,"((""diabetes, gestational""[MeSH Terms] OR (""diabetes""[All Fields] AND ""gestational""[All Fields]) OR ""gestational diabetes""[All Fields] OR (""gestational""[All Fields] AND ""diabetes""[All Fields] AND ""mellitus""[All Fields]) OR ""gestational diabetes mellitus""[All Fields]) AND (""diet""[MeSH Terms] OR ""diet""[All Fields] OR ""dietary""[All Fields] OR ""dietaries""[All Fields]) AND (""inflammatories""[All Fields] OR ""inflammatory""[All Fields]) AND (""abstracting and indexing""[MeSH Terms] OR (""abstracting""[All Fields] AND (y_10[Filter])"
EMBASE	(‘gestational diabetes mellitus’/exp OR ‘gestational diabetes mellitus’ OR (gestational AND (‘diabetes’/exp OR diabetes) AND mellitus)) AND (‘dietary inflammatory index’/exp OR ‘dietary inflammatory index’ OR (dietary AND inflammatory AND (‘index’/exp OR index))) AND [2014-2025]/py
GOOGLE SCHOLAR	allintitle: Gestational Diabetes Mellitus and Dietary Inflammatory Index, dates: 2014 and 2024
BVS	gestational diabetes mellitus AND dietary inflammatory index AND (year_cluster:[2014 TO 2024])

Initially, titles and abstracts were read. Subsequently, the full text was evaluated regarding the study type, dietary characteristics, number of parameters based on data from Shivappa et al. ^(18)^ (Table S1), and outcomes related to pregnant women with GDM (primary outcome).

The primary outcome was related to the association between DII and the risk of GDM. Secondary outcomes were related to the prevalence of GDM and other associated risk factors.

The risk of bias was assessed using the Quality Assessment Tool according to the evaluation type: type 1 (cross-sectional studies) and type 2 (case-control). The tools contained between 12 and 14 questions, where the quality could be classified as "0" for poor (up to 30%), "i" for fair (30 to 69%), or "ii" for good (>70).^(22)^

The data synthesis will be written descriptively and narratively, presenting the year, study design, population, sample size, location, objective, methods of dietary recall evaluation, dietary inflammatory index assessment, and outcomes of the selected articles.

## Results

**Figure 1:**
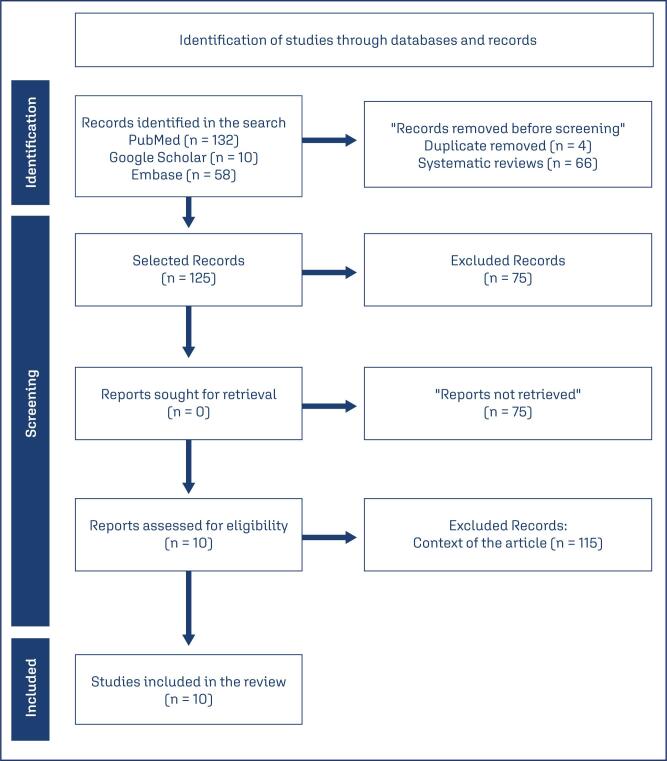
Flowchart of study screening based on the PRISMA Guidelines

Two hundred studies were screened, and those that did not meet the inclusion criteria, duplicates, and those that, after full analysis, met the exclusion criteria were removed. Ten studies that analyzed the relationship between DII and the risk of GDM were then evaluated (Figure 1).

More studies were published from 2021 (60%), with China as the country of origin (40.0%) and cohort design (60.0%). It was also observed that 60% of the included studies used the Food Frequency Questionnaire for dietary assessment, with significant variation in the number of parameters evaluated for calculating the DII, where 30% used 28 parameters, 60% of the studies evaluated DII in tertiles or quartiles, and 80.0% of the studies evaluated DII without adjustment for energy intake (Chart 2).

**Chart 2:** Characterization of the evaluated studies

Author	Year	Country	Study Type	Diet assessment	GDM diagnosis	DII parameters	Adjustment for energy intake
Zhang et al.^(10)^	2021	China	Cohort	FFQ	Glucose Tolerance	26	No
Pajunen et al.^(16)^	2022	Finland	Case - control	Food Diary	Glucose Tolerance	28	Both
Shivappa et al.^(19)^	2019	Iran	Case - control	FFQ	Glucose Tolerance	32	No
Sen et al.^(21)^	2016	USA	Cohort	FFQ	Glucose Tolerance	28	No
Zeng and Piao^(23)^	2024	China	Case - control	R24	Medical Report	28	No
McCullough et al.^(24)^	2017	USA	Cohort	FFQ	Medical Report	27	Yes
Kyozuka et al.^(25)^	2022	Japan	Cohort	FFQ	Glucose Tolerance	30	No
Soltani et al.^(26)^	2021	Iran	Cohort	FFQ	Glucose Tolerance	29	No
Wu et al.^(27)^	2021	China	Case - control	FFQ	Glucose Tolerance	17	No
Zhao et al.^(28)^	2018	China	Cohort	R24	Glucose Tolerance	20	No

FFQ: Food Frequency Questionnaire; GDM: Gestational Diabetes Mellitus; DII: Dietary Inflammatory Index; FFQ: Food Frequency Questionnaire; R24: 24-hour recall

As shown in chart 3, most studies evaluated factors associated with DII and the relationship with the risk of GDM (90.0%). Conversely, one study aimed to evaluate DII with adverse pregnancy outcomes, also presenting data on GDM in its analysis.

**Chart 3:** Titles, objectives, and comparisons of the evaluated studies.

Author	Title	Objective	Comparisons
Zhang et al.^(10)^	Association between dietary inflammatory index and risk of gestational diabetes mellitus in a prospective cohort study of births.	Investigate the association between the inflammatory potential of the diet, measured by the dietary inflammatory index (DII), and the risk of gestational diabetes mellitus (GDM) in pregnant Chinese women.	DII Terciles
Pajunen et al.^(16)^	A healthy dietary pattern with low inflammatory potential reduces the risk of GDM	Investigate the contribution of diet to the development of GDM in a comprehensive manner	GDM Yes and No
Shivappa et al.^(19)^	Association between the inflammatory potential of the diet and the likelihood of gestational diabetes mellitus among Iranian women	To examine the association between DII scores and GDM in a case-control study conducted in Iran	GDM Yes and No
Sen et al.^(21)^	The inflammatory potential of the diet during pregnancy is associated with lower fetal growth and breastfeeding failure: results from the Viva Project	To examine associations of the maternal prenatal DII, a composite measure of the inflammatory potential of the diet, with markers of maternal systemic inflammation and pregnancy	DII Quartiles
Zeng and Piao^(23)^	Association analysis of dietary inflammatory index and gestational diabetes mellitus: based on the national health and nutrition survey database	Associate the DII with the risk of GDM	GDM Yes and No
McCullough et al.^(24)^	Maternal inflammatory diet and adverse pregnancy outcomes: circulating cytokines and genomic imprinting as potential regulators?	To examine the association between maternal DII and child birth outcomes	DII Quartiles
Kyozuka et al.^(25)^	Preconception dietary inflammatory index and risk of gestational diabetes mellitus based on maternal body mass index: results from a Japanese birth cohort study	To examine the impact of a pre-conceptional pro-inflammatory diet on GDM	DII Quartiles
Soltani et al.^(26)^	Association between dietary inflammatory potential and risk of developing GDM: a prospective cohort study	To assess the association between the DII score during the first trimester of pregnancy and the risk of developing GDM among Iranian women	DII Quartiles
Wu et al.^(27)^	Correlation between dietary inflammatory index and neutrophil/lymphocyte ratio in patients with GDM	Understand the relationship between the DII and gestational diabetes, and the ratio of DII to neutral collectors/lymphatic cells, blood sugar	GDM Yes and No
Zhao et al.^(28)^	Prospective associations of dietary inflammatory index and high-sensitivity serum C-reactive protein in the second trimester of pregnancy with the risk of GDM	To evaluate the efficacy of the dietary DI in pregnant women and the association between DII and high-sensitivity serum C-reactive protein in the second trimester of pregnancy and the DM).	DII Terciles

As shown in chart 4, the number of pregnant women in the studies ranged from 164 to 90,740. Excluding studies that conducted assessments with a predefined control group, the incidence of GDM varied between 2.7 and 28.4%.

**Chart 4:** Incidence of GDM by study

Author	n	GDM n(%)
Zhang et al.^(10)^	2639	347 (13.1)
Pajunen et al.^(16)^	351	-
Shivappa et al.^(19)^	388	-
Sen et al.^(21)^	1692	96 (5.4)
Zeng and Piao^(23)^	1421	137 (9.6)
McCullough et al.^(24)^	1057	64 (6.0)
Kyozuka et al.^(25)^	90740	2405 (2.7)
Soltani et al.^(26)^	812	231 (28.4)
Wu et al.^(27)^	164	-
Zhao et al.^(28)^	336	43 (12.8)

GDM: Gestational Diabetes Mellitus

Of the evaluated studies, 4 compared cases with and without GDM, observing higher DII values in the GDM group (Chart 5).

**Chart 5:** Mean DII values in patients with and without GDM among the case-control studies evaluated

Author	Without DMG	With DMG	p-value £
Pajunen et al.^(16)^	− 1.33 ± 1.56	− 0.70 ± 1.59	0.002
Shivappa et al.^(19)^	−0.07 ± 1.05	0.15 ± 0.89	0.040
Zeng and Piao^(23)^	1.55 ± 1.74	1.94 ± 1.53	0.010
Wu et al.^(27)^	-0.85 ± 0.45	-0.51 ± 0.83	<0.001

DII: Dietary Inflammatory Index; GDM: Gestational Diabetes Mellitus.

£ Student's t-test

Six studies compared the incidence of GDM according to DII tertiles or quartiles, with only one study observing a higher proportion of patients with GDM in the highest DII tertile (p= 0.006), while the others did not present significant differences (Chart 6).

**Chart 6:** Presence of GDM among terciles or quartiles in the cohort studies evaluated

Author	1st DII	2nd DII	3th DII	4th DII	p- value ¥
Zhang et al.^(10)^	101 (11.5)	122 (13.9)	124 (14.1)		0.202
Sen et al.^(21)^	25 (5.6)	30 (6.7)	27 (6.1)	14 (3.1)	0.902 *
McCullough et al.^(24)^	18 (7.0)	16 (6.0)	13 (5.0)	17 (7.0)	0.808
Kyozuka et al.^(25)^	613 (2.7)	615 (2.7)	614 (2.7)	563 (2.5)	0.256
Soltani et al.^(26)^	248 (30.6)	239 (29.4)	202 (24.9)	253 (31.1)	0.500
Zhao et al.^(28)^	6 (5.36)	15 (13.39)	22 (19.64)		0.006

DII: Dietary Inflammatory Index;

¥ Chi-square/Fisher's Exact test

* Calculation performed using data available from the study.

Among studies that tested models adjusted for anthropometry, gestational history, and dietary intake, 60% evaluated the presence of an association between DII and the risk of GDM. Additionally, in 60% of the studies, these adjustment variables were related to the risk of GDM, although one study found no significant relationship between them and the risk of GDM (Chart 7).

**Chart 7:** Factors associated with DII and GDM

Author	Risks associated with DII+GDM in adjusted models	Other associations with GDM
Zhang et al.^(10)^	Total energy intake, gestational week in the FFQ, weight gain before GDM diagnosis, and use of multivitamin supplements	Pre-gestational overweight or obesity
Pajunen et al.^(16)^	Pre-gestational BMI and intervention groups	Higher pre-gestational BMI and diastolic blood pressure, greater intake of total fat, trans fatty acids, and zinc, and lower fiber consumption
Shivappa et al.^(19)^	Age and energy consumption	Higher BMI and family history of diabetes, and lower gestational age and physical activity
Sen et al.^(21)^	Excess weight	Not assessed
Zeng and Piao^(23)^	Age, race, BMI, smoking, alcohol consumption, hypertension, history of cardiovascular events, arthritis, platelet count, neutrophils, lymphocytes, white blood cells, monocytes, eosinophils, and basophils	Not assessed
McCullough et al.^(24)^	Not assessed	Not assessed
Kyozuka et al.^(25)^	Pre-gestational BMI	Higher gestational BMI
Soltani et al.^(26)^	No associations even with adjustment	Not assessed
Wu et al.^(27)^	Not assessed	Lower HDL and consumption of inflammatory nutrients, higher carbohydrate and iron intake, education level
Zhao et al.^(28)^	Not assessed	Lower HDL and consumption of inflammatory nutrients, higher carbohydrate and iron intake, education level.

DII: Dietary Inflammatory Index; GDM: Gestational Diabetes Mellitus; FFQ: Food Frequency Questionnaire; BMI: Body Mass Index; HDL: High Density Lipoprotein

The studies were evaluated concerning the level of bias and research quality, with 90.0% of the works considered of good quality according to the criteria of the National Heart, Lung, and Blood Institute (Chart 8).

**Chart 8:** Risk of bias and quality of the studies

Author	Assessment	Q1	Q2	Q3	Q4	Q5	Q6	Q7	Q8	Q9	Q10	Q11	Q12	Q13	Q14	Quality
Zhang et al.^(10)^	Cohort	Y	Y	Y	Y	N	Y	NA	Y	Y	NA	Y	N	Y	Y	ii
Pajunen et al.^(16)^	Case – control	Y	Y	Y	Y	Y	Y	CD	N	Y	S	N	N	-	-	ii
Shivappa et al.^(19)^	Cohort	Y	Y	Y	Y	Y	Y	NA	Y	Y	NA	Y	N	Y	Y	ii
Sen et al.^(21)^	Cohort	Y	N	CD	CD	CD	Y	NA	Y	Y	NA	Y	N	Y	Y	i
Zeng and Piao^(23)^	Cohort	Y	Y	Y	Y	N	Y	NA	Y	Y	NA	Y	N	Y	Y	ii
McCullough et al.^(24)^	Cohort	Y	Y	Y	Y	Y	Y	NA	Y	Y	NA	Y	N	Y	Y	ii
Kyozuka et al.^(25)^	Cohort	Y	Y	Y	Y	Y	Y	NA	Y	Y	NA	Y	N	Y	Y	ii
Soltani et al.^(26)^	Case – control	Y	Y	Y	Y	Y	Y	CD	N	Y	S	N	N	-	-	ii
Wu et al.^(27)^	Case - control	Y	Y	Y	Y	Y	Y	CD	N	Y	S	N	N	-	-	ii
Zhao et al.^(28)^	Case - control	Y	Y	Y	Y	Y	Y	CD	N	Y	S	N	N	-	-	ii

CD: Cannot determine; NA: Not applicable

## Discussion

In our review, we systematically investigated 10 studies that evaluated the relationship between the DII and the risk of GDM) The results showed a variation in the diagnosis of GDM from 2.7% to 28.4%. We identified four case-control studies,^(16,19,23,27)^ whose results showed an increased DII score in the group of pregnant women with GDM compared to those without a diagnosis. Analyzing the other cohort studies,^(10,21,24–26,28)^ that compared the incidence of GDM according to DII tertiles and quartiles, we observed that only one study reported a higher proportion of pregnant women with GDM in the third tertile.^(28)^ Additionally, this review showed an association between DII and the risk of GDM in five studies that used models adjusted for anthropometry, gestational history, and dietary intake.^(10,21,24–26)^

Studies have shown that a Mediterranean dietary pattern reduces the incidence of GDM and inflammation in pregnant women due to its anti-inflammatory properties.^(29)^ Our findings demonstrated a variation in disease diagnosis, which may be primarily related to the nutritional status of these women, as most of them had obesity, as described in the analyzed studies. An important observation in our study was regarding the standardization of GDM diagnosis. Among the ten selected studies, two used previous medical reports for GDM diagnosis,^(23,24)^ while the others used the Glucose Tolerance Test (GTT). These findings align with literature descriptions that estimate the prevalence of GDM to vary between 8.4% and 30.9%, with this variation associated with different diagnostic methods and population characteristics.^(6,7)^

Various studies describe the mechanism of action of a pro-inflammatory diet in the risk of GDM.^(30–32)^ Conversely, an anti-inflammatory diet is inversely associated with a reduction in the occurrence of the disease.^(29)^ In our review, the highest DII was found in pregnant women with GDM in case-control studies, primarily associated with the type of diet consumed and nutritional status. These results are consistent with data indicating that high consumption of refined grains, sugars, fats, red meats, processed foods, and low consumption of fruits, legumes, and vegetables is associated with increased inflammation and risk of GDM.^(33)^

A pro-inflammatory diet increases blood levels of inflammatory substances such as CRP, TNF-α, and IL-6, consequently increasing the body's inflammation degree.^(34,35)^ Furthermore, studies have shown that the consumption of processed and unprocessed red meats is also associated with a higher risk of developing GDM during pregnancy, even after adjustments for body mass index (BMI).^(36,37)^ These findings align with our results, as we identified an association between high DII and GDM risk from models adjusted for anthropometry, gestational history, and dietary intake.

Scientific evidence also demonstrates the importance of a healthy gut microbiota in reducing body inflammation.^(38)^ Zheng et al.,^(39)^ investigating the relationship between DII and gut microbiota, observed that the pro-inflammatory diet group had elevated levels of *Ruminococcus torques*, *Eubacterium nodatum*, and *Clostridium leptum* compared to the anti-inflammatory diet group.^(39)^ These findings demonstrate the relationship between pro-inflammatory diet components and gut microbiota imbalance.

In this study, we observed an association between increased DII and the risk of GDM. However, the review has some limitations. First, we identified a lack of standardization in dietary intake assessment questionnaires. Three different methods were identified for evaluating the diet consumed by pregnant women, which may have impacted the different DII score results. The second limitation was the difference in parameters used to evaluate DII in pregnant women. Shivappa et al.^(18)^ proposed using 45 parameters to define the inflammatory potential of a diet. In the evaluated studies, we observed variation in the number of parameters used to determine the DII of pregnant women. Consequently, the lack of use of all 45 parameters in the found studies may have affected the DII score results in classifying the diet as pro-inflammatory or anti-inflammatory. The third limitation of our review is associated with the study model used. Among the ten studies, six were cohort studies, and only four were case-control studies. New case-control studies are needed using the 45 parameters proposed by Shivappa et al.^(18)^ to determine the inflammatory effect of the diet on pregnant women.

## Conclusion

This systematic review revealed that pregnant women diagnosed with Gestational Diabetes Mellitus had a more pro-inflammatory diet compared to those without the diagnosis during pregnancy. The DII can be used as an important risk parameter for Gestational Diabetes Mellitus when also considering the nutritional status and history of the pregnant woman.
